# Methods used in microbial forensics and epidemiological investigations for stronger health systems

**DOI:** 10.1080/20961790.2021.2023272

**Published:** 2022-03-23

**Authors:** Arizaldo E. Castro, Maria Corazon A. De Ungria

**Affiliations:** a Microbial Ecology of Terrestrial and Aquatic Systems Laboratory, Institute of Biology, University of the Philippines Diliman, Quezon City, Philippines; b DNA Analysis Laboratory, Natural Sciences Research Institute, University of the Philippines Diliman, Quezon City, Philippines; c Program on Biodiversity, Ethnicity, and Forensics, Philippine Genome Center, University of the Philippines Diliman, Quezon City, Philippines

**Keywords:** Forensic sciences, microbial forensics, epidemiology, infectious diseases, molecular methods, health system resilience, microbial forensic systems

## Abstract

This review discusses microbial forensics as an emerging science that finds application in protecting human health. It is important to distinguish naturally acquired infections from those caused by the intentional release of microorganisms to the environment. This information is crucial in formulating procedures against the spread of infectious diseases and prosecuting persons who may be involved in acts of biocrime, bioterrorism, or biowarfare. A comparison between epidemiological investigations and microbial forensic investigations is provided. In addition, a discussion on how microbial forensics strengthens health systems is included in this review. Microbial forensic investigations and epidemiologic examinations employ similar concepts and involve identifying and characterising the microbe of interest. Both fields require formulating an appropriate case definition, determining a pathogen’s mode of transmission, and identifying the source(s) of infection. However, the two subdisciplines differ in their objectives. An epidemiological investigation aims to identify the pathogen’s source to prevent the spread of the disease. Microbial forensics focuses on source-tracking to facilitate the prosecution of persons responsible for the spread of a pathogen. Both fields use molecular techniques in analysing and comparing DNA, gene products, and biomolecules to identify and characterise the microorganisms of interest. We included case studies to show methods used in microbial forensic investigations, a brief discussion of the public significance of microbial forensic systems, and a roadmap for establishing a system at a national level. This system is expected to strengthen a country’s capacity to respond to public health emergencies. Several factors must be considered in establishing national microbial forensic systems. First is the inherent ubiquity, diversity, and adaptability of microorganisms that warrants the use of robust and accurate molecular typing systems. Second, the availability of facilities and scientists who have been trained in epidemiology, molecular biology, bioinformatics, and data analytics. Human resources and infrastructure are critical requirements because formulating strategies and allocating resources in times of infectious disease outbreaks must be data-driven. Establishing and maintaining a national microbial forensic system to strengthen capacities in conducting forensic and epidemiological investigations should be prioritised by all countries, accompanied by a national policy that sets the legislative framework and provides for the system’s financial requirements.

## Key points

Microbial forensics and molecular epidemiology employ similar molecular analytical techniques in studying microbial infectious agents. Still, they differ in the rigor of standard, the requirement for a chain of custody of samples, and the strength of accredited operational procedures.

National microbial forensic systems are envisioned to monitor incidents of microbial infections in a given population. Biosurveillance takes notice of emerging infectious diseases and those already controlled previously but are re-emerging in the population.

National microbial forensic systems must address the challenges related to the inherent diversity of microorganisms, the requirement for robust molecular typing systems, and the availability of scientists with technical expertise in microbial forensics, molecular biology, bioinformatics, and data analytics.

## Introduction

Microbial forensics is an emerging field that combines microbiology and forensic science [1–4]. It has its origins in molecular epidemiology [5, 6]. Both fields involve the study of “outbreak” events to understand the extent and the transmission of individual microbes across the affected population. Applying genetic, chemical, and physical information, scientists use physical, biochemical, and molecular tests to identify and characterise the microbes of interest. Reliable characterisation of molecular variations between related strains identifies microorganisms down to the substrain level [2–4, 7]. In microbial forensics, the accurate identification of the microbial agent to the substrain level can contribute to identifying the person responsible for the attack or the so-called “attribution” process [8, 9]. Although both fields share methodologies, microbial forensics has the added requirement that its protocols withstand legal scrutiny. Hence, microbial forensic scientists must also be trained in evidence collection, handling and storage, and expert testimony. The results of microbial forensic investigations are likely to be used as evidence to support or negate an allegation in court, which necessitates exhaustive and independent testing as well as unbiased systems to achieve maximum sensitivity and specificity [7, 10, 11].

Bacteria in the human body have an uneven distribution, with each site—skin, dental plaque, saliva, stomach, duodenum, jejunum, ileum, colon—showing specific taxonomic compositions. The presence and distribution of microbes in the human body are influenced by genetics, age, diet, personal hygiene, antimicrobial drugs, and environmental exposure. The use of site-specific microbiomes or specific microorganisms for human identification requires substantive studies that assess microbial stability and spatial-temporal differentiation per individual under predefined conditions[12]. For example, the high variability of skin microbiomes could be used to distinguish persons present in a place at a specific time. Directed polymerase chain reaction (PCR) and metagenomic sequencing of skin microbiomes increase the capacity to identify human sources of samples in forensic investigations [13]. *Cutibacterium acnes* (formerly known as *Propioniobacterium acnes*), responsible for acne development, is an example of a bacterial species used in microbial forensics. It possesses strain-specific single nucleotide variations per individual [14] and may, therefore, be considered a potential forensic target using trained large-scale logistic models [15]. In addition, studies on determining the time of death, identifying the cause of death, moving bodies from one site to another, postmortem interval (PMI) estimation, and establishing links between personal items and persons of interest are available [16]. Several bacterial families, genera, and species are useful for PMI estimation (*Actinomycetaceae*, *Bacteroidaceae*, *Alcaligenaceae*, and *Bacilli*), for cause of death identification (*Aeromonas*), and geolocation (*Corynebacterium* or *Helicobacter pylori*) [16].

Microbial forensics should be an integral part of forensic investigations to determine if a biocrime, bioterrorism, or biowarfare has been committed, especially in covert cases [5, 6]. Biocrime is the intentional use of biological agents against a specific individual [1]. In contrast, bioterrorism that may be motivated by political or religious ideologies uses destructive biological agents to harm a population [1, 17]. The development, production, and use of chemical and biological agents in warfare were recognised as threats to global public safety as early as the 1960s [17]. In the United States, two events that showed the critical contribution of microbial forensics to counter-terrorism are the 2001 anthrax mailings [6] and the September 11 terrorist attack or the “9/11 tragedy” [18]. These attacks focused the world’s attention on the need to protect responders, the proper management of the “crime scene”, and the requirement of the chain of custody of a large number of samples that could be used by the court to decide on the guilt or innocence of any individual suspected of “authoring” the attack. Because of the complexity and scale of the analysis, scientists in different fields—microbiologists, molecular biologists, and computational scientists—were provided with dedicated equipment and infrastructure to process and analyse samples recovered from Ground Zero [7]. The 2001 anthrax mailings led the US Department of Homeland Security to establish the National Bioforensics Analysis Center (NBFAC) in 2004 [6, 19]. Since then, the NBFAC has worked with the Federal Bureau of Investigation (FBI) in assessing intelligence and responding to threats using high-level analysis of samples that may be potential threats to the population [20]. The NBFAC is a central facility that processes samples using the best scientific resources with fully accredited, state-of-the-art lab facilities up to Biosafety Level (BSL) 4, which allows it to handle pathogens that have no existing vaccine or treatment [19, 20]. In many countries, establishing centralised NBFAC-like facilities dedicated to microbial forensics and epidemiological investigations must be seriously considered due to the increasing number of novel and emerging human pathogens [5, 12].

This review discusses how microbial forensics and molecular epidemiology should be used to understand the nature and adaptability of infectious microbes, which permit them to endure selective pressures in the environment. We argue for the importance of microbial forensics in answering legal questions such as those associated with recent anthropogenic events that affected communities and nations, e.g. disease outbreaks and biological attacks.

## The burden of microbial infections

For the longest time, infectious diseases have been the major cause of premature death and disability worldwide. Non-communicable diseases eventually replaced infectious diseases from the top post because of increased sanitation, improved water supply, vaccine, and drug development. However, the effects of these developments vary across the world, with high-income countries (HICs) showing significantly decreased mortality rates due to infectious diseases. In contrast, lower- and middle-income countries (LMICs), with limited resources, continue to face the problems caused by infectious microbes in high proportions of their populations [21].

A pathogen must be able to colonise a host, thrive, and replicate in a specific niche in the host’s body (tissue tropism) while avoiding attacks from the host’s innate and adaptive immune system. Microbes express virulence genes for their protection and growth within the host, leading to disease pathogenesis and resulting in the host’s death if the infection is not properly managed. Meanwhile, the infectious agent would have produced progenies transferred to new hosts, ensuring their perpetuation. Pathogen transmission is either simple, e.g. direct transmission from one host to another, or complex in which different animal or insect vectors and focal hosts are involved.

## Epidemiological investigations and microbial forensics

Animal infections and human zoonoses naturally occur. Some human activities may lead to the accidental release of biological threats to the environment. Table 1 lists the workflow for applying molecular epidemiology in investigating outbreaks of naturally occurring infectious diseases and how microbial forensics would determine if someone intentionally planned the event, thus redefining the incident as a biocrime or bioterrorist attack. In epidemiological investigations, the identification of the microbial agent provides vital information about its drug susceptibility, its source, the routes or pathways of transmission, and any other factor that can enhance transmission. In most cases, the strain under investigation is compared to strains from reference sources. The primary assumption in epidemiologic studies is that the disease is naturally occurring [5, 11]. Hence, no further work is conducted to identify persons or groups that could have intentionally caused the outbreak or the so-called “attribution” stage, an integral step in a microbial forensic investigation. On the other hand, accurate attribution is a primary objective in microbial forensics—in-depth identification of the microbial agent down to the sub-strain level is made whenever possible [11]. Laboratory manipulation of microorganisms (e.g. cloning and genetic engineering) will leave traces in their genomes. These genotypic signals as well as phenotypic features can be used to prove the deliberate production and release of harmful infectious agents to inflict harm. Investigators will also use the actual geographical location of the crime and evidence obtained from other forensic disciplines (e.g. PMI estimations) [22] to reconstruct the crime and identify the persons involved.

**Table 1.** Common steps of workflow and interaction between microbial forensics and molecular epidemiology during initial investigations.

Steps	Microbial forensics	Molecular epidemiology
**1. Collection of specimen**	Enforcement of chain of custody Follows standard operating procedures (SOPs) for sampling Lawful and accredited personnel are involved in specimen collection	More research-oriented Utilises active or passive surveillance samples
**2. Recognition that an attack or outbreak/disease is happening/** **spreading**	Requires inputs from healthcare professionals for case definition Answers questions pertinent to: Pathogen’s route of transmission and survivability (airborne, waterborne, vector-borne, persistence on surfaces, etc.) Pathogen’s virulence (comparison with control specimens and detection of virulence factors/enhanced phenotypes/strains) Appropriate control strategy (is there enough time to quarantine or eradicate)	
**3. Validation for quality assurance and control**	Establishes limits and sensitivity of methods and data collected to ensure replicability	
**4. Analysis of collected specimen**	Application of established workflows to collect relevant data (genetic, physical, and chemical) Performed in dedicated laboratories that follow ISO-accredited operating protocols with established chain of custody	Application of established workflows to collect relevant data (genetic, physical, and chemical) Performed in research/academic laboratories that do not necessarily follow stringent SOPs/accreditation

Table 2 summarises the distinct features of epidemiological investigations and microbial forensic investigations. Both are primarily concerned with source-tracking but with different minor outputs and end goals [11]. In microbial forensic investigations, the secondary purpose is to identify and prosecute perpetrators. Regular epidemiological investigations target to control the disease and identify contributing factors to prevent its recurrence [11]. Stringent protocols on sample handling and transfer are not usually present in regular epidemiologic disease outbreak assessments. Two case studies are described here to show the differences between epidemiological investigations and microbial forensic investigations [24, 25].

**Table 2.** Salient features of epidemiological investigations and microbial forensics.

Points of comparison	Epidemiological investigations	Microbial forensic investigations
**Primary goal**	Source-tracking of the pathogen to ensure prevention of the transmission and spread of disease [5, 11]	Source-tracking of the pathogen to accomplish attribution [5, 6]
**Use of molecular methodologies**	Present, molecular biotechniques that derive information from nucleic acids, proteins, etc. [5, 12]	Present, molecular biotechniques that derive information from nucleic acids, proteins, etc. [5, 12]
**Use of traditional forensic methods**	Absent [15]	Present, especially in cases of covert biocrime or bioterrorism (fingerprinting, trace material analysis, handwriting analysis) [15]
**Standard of evidence production**	Techniques used should be validated, peer-reviewed, and accepted by the relevant scientific community [7, 10, 11]	Techniques used should be validated, peer-reviewed, and accepted by the relevant scientific community; Chain of custody is strictly observed [7, 10, 11]
**Operates within a legal framework?**	No [7, 10, 11]	Yes, country-specific and international (at the level of the United Nations Security Council if multiple geographical locations are involved) [7, 10, 11, 23]

*Case 1*: Grad *et al.* [24] analysed the May to July 2011 bloody diarrhoea and hemolytic uremic syndrome (HUS) outbreak in Germany and France. Germany recorded 4 000 cases of bloody diarrhoea and 850 cases of HUS, while France had 15 cases of bloody diarrhoea, with nine cases proceeding to HUS. Both outbreaks were attributed to a strain of Shiga toxin-producing *Escherichia coli* serotype O104:H4. Genetic differences between the bacterial isolates associated with the two outbreaks were identified by sequencing 17 strains of *E. coli* O104:H4 using multiple platforms (i.e. Illumina, 454, and PacBio). Fifteen strains were sequenced from the 2011 outbreak, composed of four strains from Germany and 11 from France. Additionally, two reference strains from France that were isolated in 2004 and 2009 were also sequenced. Whole genome sequence (WGS) data with high coverage (146X) identified 21 single nucleotide polymorphisms (SNP) that were used for Maximum Likelihood phylogenetic analysis. Results showed that the German isolates were less diverse amongst themselves, with genetic differences of the four isolates detected in only two SNPs out of the entire *E. coli* genome, compared to the 19 SNPs manifested by the French isolates. The 2011 German *E. coli* O104:H4 isolates clade within the French isolates caused the 2011 outbreak rather than the 2004 and 2009 reference strains [24]. These genetic differences were validated through the analysis of WGS data and were in concordance with the standing hypotheses related to the French and German outbreaks. The diversity of the German O104:H4 isolates was probably lessened due to a bottleneck event. The mutation rates of the French and the German outbreak isolates differed significantly, and there was an uneven distribution of genetic diversity in seed populations that led to the outbreaks. The epidemiological investigation ended after the report on the phylogenetic relationship of the isolates came out.

*Case 2*: An example of applying microbial forensics to trace a pathogen was done in a criminal case in Lafayette, Louisiana, in 1994. A doctor was accused of mixing blood from two patients, one with human immunodeficiency virus-1 (HIV-1) and another with hepatitis C (HCV). The mixed blood was used to infect his former girlfriend. Metzker *et al.* [25] investigated the HIV-1 strains of the doctor’s patient and his girlfriend following a strict chain of custody throughout the investigation. The analysts did not reveal sample identity during the process, and experimental data used for phylogenetic analysis were produced in two independent laboratories. Both laboratories generated sequence data of the 858-bp *env* gene fragment and 1147-bp reverse transcriptase gene fragment of the coded samples. Phylogenetic sequence analysis showed that the girlfriend’s HIV-1 sequences were closely related to and nested with the patient’s HIV-1 sequences. Sequence-based phylogenetic analysis provided the link between the HIV-1 strains found in the patient and the girlfriend, with the medical doctor being the only connection between them. The prosecution used the analysis results to accuse the doctor of using his patient’s HIV-1 virus to infect his girlfriend, now the “victim” [25]. The microbial forensic investigation ended with the successful attribution of the biocrime to the doctor. The doctor was convicted of attempted second-degree murder [25]. The paper did not include information on the characterisation of the HCV initially alleged to have been isolated by the doctor from another patient and added to the injected mixture.

## Molecular methods in studying microbes

Numerous techniques such as phenotypic assays based on the physiological properties of different microbial agents, characterisation of biomolecules derived from pathogens, gene expression analyses, and DNA sequencing have been used to identify microbes [5, 12]. Summarised comparison of molecular methods commonly used in microbial forensics and epidemiological investigations is provided in Table 3.

**Table 3.** Comparison among molecular techniques employed in microbial forensics and molecular epidemiology.

Molecular techniques	Advantages/strengths	Disadvantages/weaknesses
**Nucleic acid sequence analyses and fingerprinting**	Uses a stable biomolecule as the data source, i.e. DNA Analyses can be carried out without direct manipulation of DNA, *at the minimum DNA extraction and shearing in DNA fingerprinting*	Analyses dependent on quantity and quality of extracted DNA Database-dependent—analyses involving novel sequences/fingerprints may be inconclusive
**Molecular phylogeny**	Applicable to present and historical (banked) samples Could be used in deriving supplementary information in support of taxonomy assignment	Interpretation of resulting outputs will require advanced technical expertise Database-dependent—if reference sequences are limited, analyses and potential inferences are affected
**Gene expression**	Complements/supplements genotype, biochemical, and chemotaxonomic data Helpful in understanding the physiology of pathogens	Costly and may not be suitable in resource-limited settings, i.e. LMICs Needs comprehensive understanding of the role of the gene of interest in microbial metabolism
**Microbe-derived biomolecule analyses**	Could serve as direct evidence, i.e. bacteria-derived toxins Could be used in deriving supplementary information in support of taxonomy assignment	Costly and may not be suitable in resource-limited settings, i.e. LMICs Database-dependent—limited analyses if the database is not all-encompassing or is not regularly updated

LMICs: lower- and middle-income countries.

### Nucleic acid analyses

Several DNA markers are extremely useful in microbial identification [19]. High-throughput sequencing (HTS) techniques or next-generation sequencing (NGS) methods have been used to assign characteristics to a microorganism without the need to conduct biochemical and phenotypic assays. Taxonomic classification of microbes is very useful in differentiating closely related strains or isolates due to the availability of all information about an organism and the absence of inherent bias during the analysis [7, 26, 27]. Unlike multilocus sequence typing (MLST) that uses smaller DNA regions (genes, operons, or chromosomes), WGS-based taxonomy assignment uses substantial genotype data in order to identify microorganisms down to the substrain level [27].

The availability of robust sequencing technologies has opened possibilities for microbial forensics and epidemiological investigations. There is interest in studying the feasibility of using skin microbiota to identify its human source. Using *C. acnes* as the study system, Yang *et al.* [28] devised a high-throughput genotyping method to characterise the SNPs found in the 16S rRNA gene. Interestingly, *C. acnes* genotypes were found to be individual-specific. Combining skin microbiome profile and *C. acnes* 16S rRNA genotype was reported to provide as high as 90% accuracy in DNA source/host identification. However, before adopting skin microbiome characterisation as part of forensic investigations, the following considerations must be addressed: (1) long-term instability of skin microbiota, (2) the effect of contamination with transient skin microbes, (3) effect of the environment, and (4) reproducibility of results. Once these limitations are addressed, the proposed method is potentially novel with high precision and reproducibility [28].

The use of WGS data has expedited the timely identification of microorganisms and their variants after prolonged infection and widespread community transmissions. In the current global pandemic, SARS-CoV-2 variants have been identified and linked to accurately document the spread of the virus within and across national borders. The G614 SARS-CoV-2 variant from Europe was detected in patient samples in Pakistan using WGS data [29]. Bar-Or *et al.* [30] identified the presence of the B.1.1.7 strain or the so-called alpha variant in Israel wastewater samples which corresponded with clinical data in December 2020. The B.1.1.7 variant originally detected in the UK in September 2020 was also found in localities where not enough clinical sampling had been done, thus alerting the government to the need for a more inclusive biosurveillance protocol. The World Health Organization has identified three additional SARS-CoV-2 variants of concern. These variants are the beta variant (B.1.351) reported in South Africa in May 2020, the gamma variant (lineage P.1) that came out in Brazil in November 2020, and the delta variant (B.1.617.2) that have originated in India and reported in October 2020 [31]. The delta variant’s ability to infect a patient without immediately manifesting itself and remaining undetected has been key in its extremely rapid spread in India, the Philippines, USA, Australia, and many other countries.

### DNA fingerprint analyses

Multiple locus variable number tandem repeat analysis (MLVNTRA) is another technique used to produce the DNA fingerprint of a microbial isolate. In MLVNTRA, microbial DNA regions known as variable number tandem repeats (VNTRs) are amplified by PCR. The sizes of the VNTRs are determined and converted into allele types. The allele types of a strain under investigation will be compared to known reference strains to facilitate identification. Multiple genetic variations could indicate high diversity among different strains in a single taxonomic unit, or the microbial agent of interest may have been misclassified [32]. To test the method, Yun *et al.* [33] used a combination of MLVNTRA with capillary electrophoresis to characterise the genetic relationship and population structure of 31 *E. coli* O157:H7 strains isolated from animal and human hosts in China. From their analysis, 29 unique MLVNTRA profiles were identified and those associated with humans were found to be unique. The group’s report backs up the high discriminatory power of MLVNTRA and hence its attractiveness as a microbial forensic technique since the equipment used is widely available and employed in the forensic community. In another study, Timmons et al. [34] developed an MLVNTRA assay to distinguish non-O157 Shiga toxin-producing *E. coli* (STEC) serogroups (i.e. O26, O111, O103, O121, O45, and O145). Highly conserved and diverse VNTR loci for intraserogroup and interserogroup discrimination were determined by the team using publicly available published genomes. The assay included 10 VNTR loci that could distinguish isolates into their respective intraserogroups and interserogroups. After PCR amplification, fragment analysis was performed to establish the MLVNTRA schemes from the isolates. The MLVNTRA schemes were concordant with pulse field gel electrophoresis (PFGE) patterns produced using *Xba*I and *Bln*I restriction enzymes. In addition, the group reported high congruence of results from MLVNTRA and PFGE with epidemiological data for all six tested serogroups, suggesting that MLVNTRA could be a versatile tool for bacterial strain differentiation.

Another case of using DNA fingerprints in microbial forensics is the study of Tims *et al.* [35]. Tims and colleagues isolated skin bacteria and tested the suitability of physical fingerprints to reveal human host information. Volunteers from Europe and Asia submitted skin samples twice. The first collection was intended to isolate transient fingertip microbiota, whereas the second collection aimed at the resident skin microbiota. PFGE DNA fingerprints of the isolates revealed that transient skin microbes are different from resident isolates. Extensive variability in microbial persistence, the time variation of the transient and resident microbiota, and the continental source of microbes, e.g. European *vs*. Asian samples, except for *Staphylococcus* strains, did not support the use of human fingertip skin for microbial forensic investigations.

### Molecular phylogeny analyses

Phylogeny involves the study of relationships among different organisms and their evolutionary development. Hence the information derived from the phylogenetic analysis has been used to define organisms as individual species. The phylogenetic species concept advocates that a species is a whole group of organisms whose members share a common ancestor and possess derived traits that distinguish them from members of other species. However, phenotype-based microbial identification is limited because of the existence of cryptic taxa and phenotypic plasticity in some species. With such challenges, contemporary taxonomy and systematics are progressively using molecular data to shift from phenotype-based to molecular. The molecular phylogenetic approach involves the reconstruction of the history of a family of homologous genes (a gene tree) that can eventually be compared to a presumed species history in which the genes are found (species tree) [36]. Prokaryotic taxonomy and phylogeny have been examined largely through analyses and comparisons of the 16S rDNA. Identification of prokaryotic operational taxonomic units (OTU) and their assignment to known taxa are based on set thresholds in genetic distances of the 16S rRNA gene ranging from 97% to 99%.

A feature in microbial DNA that is useful for molecular phylogeny analysis are SNPs. An SNP is a variation in a single position in a DNA sequence among individual organisms. As genetic markers, SNPs are associated with both coding and non-coding DNA, are highly stable and informative for lineage studies but have low power identifying individual isolates [4]. In molecular phylogeny analysis, SNPs are extremely useful in identifying microbial lineages. Certain SNPs may be chosen to represent phylogenetic branches, e.g. Canonical SNPs (canSNPs), and are definitive markers for branches and subpopulations [8]. Yan et al. [37] reported a two-step method for tracing microbial strains of public health significance, i.e. outbreak or pandemic-causing strains. The phylogenetic position of the outbreak strain in a species tree is determined, followed by a detailed description of the strain and its suspected relatives. The study compared WGS from cultured *Yersinia pestis* and historical outbreak strains to identify and select informative SNPs. These informative SNPs were used in phylogenetic analyses using the maximum likelihood, maximum parsimony, and neighbour joining methods. One thousand five hundred and sixty-four informative SNPs from 24 representative strains and seven outbreak strains produced an initial phylogenetic cluster. Afterward, 348 SNPs from 17 representative and five historical strains were used to trace the strain under investigation [37]. The authors emphasised the low computational requirement of this strategy readily available in low-resource settings. However, the need for researchers with bioinformatics experience, the requirement for extensive published WGS data from across the globe, the need to implement comprehensive comparative analysis across samples, and the absence of a unified publicly accessible database limit the immediate use of this strategy [37].

### Gene expression analyses

Microarrays are useful in determining the expression of multiple genes [38]. In microbial forensics and molecular epidemiology, microarray analysis is important to determine whether virulence genes are expressed by the microbial agent of interest [3]. Microarray technology allows identifying artificially constructed pathogens as well as those expressing drug resistance genes. Unlike nucleic acid analyses, DNA fingerprint analyses, and molecular phylogeny analyses, published literature regarding the application of gene expression analysis in microbial forensics or epidemiological investigations is not abundant.

### Biomolecule analyses

Non-nucleotide biomolecules such as proteins and carbohydrates are also useful in the characterisation of microbes because they provide information on microbial physiology [39]. Methods such as matrix-assisted laser desorption/ionization-time of flight mass spectrometry (MALDI-TOF MS), tandem mass spectrometry (TMS), and liquid chromatography (LC) are powerful tools in detecting and quantifying multiple types of biomolecules. Since extracts from bacterial cells in different life cycle phases which determine phenotype profiles can be used for biomolecule analyses, combining culturomics and genomics makes identifying bacterial species more accurate [40]. For example, Deatherage Kaiser *et al.* [41] studied the reproducibility of bacterial protein profile patterns using *Clostridium botulinum* as a study system. The effect of bacterial strain, culture medium, and the growth phase on protein expression profiles was determined using LC and TMS. The growth phase was shown to have the highest effect on protein expression.

### Quantifying the microbial agent of interest

The accuracy of bacterial identification is increased when supplemented with information derived from population and genetic diversity estimates. Cluster analysis, DNA fingerprinting, and targeted PCR quantify microbes from mixed sources and use the concept of OTU. OTUs are proxies to species identification that uses sequence data similarity [42]. In epidemiological and microbial forensic investigations, estimating the population size of a biological agent is critical in determining the type of response required, the level of protection needed for responders, how fast the potential biologic agent may spread, and the pathways of transmission.

### Source tracing: choice of technique and attribution

The sample source, e.g. food, water, soil, plants, arthropods, and vertebrates, including environmental elements in the atmosphere, must be considered in selecting microbial isolation and analysis methods. Genetic fingerprinting of microbial communities, competitive PCR, *in situ* hybridization (ISH), and PCR have been used in characterising the population dynamics of food-associated microbial communities [43]. Meanwhile, the use of ISH, fluorescent-dependent techniques, oligonucleotide microarray analysis, PCR, and restriction fragment length polymorphism have been reported for water samples [44]. Moreover, the use of culture-independent molecular techniques that do not require microbial isolation should also be considered. The increasing availability of gene, transcriptomic, and genomic sequence data helps trace sources, including viable but non-culturable strains, without the need to isolate and cultivate the microorganisms of interest [7].

Microbial forensics lacks a consensus on assessing hypotheses for source-tracking. The need for this consensus stems from the fact that in juridical hearings, easily interpreted evidence is a must because of the diversity of the audience and jury officers. Recognising this gap, Lindgren *et al.* [45] developed an approach using probabilistic evidential values, which is grounded in the likelihood ratio method. The likelihood ratio is defined as the ratio of the probability of the DNA evidence given the main hypothesis to the probability of the DNA evidence given the alternative hypothesis. Microbial forensics can follow this approach when assessing the main and the alternative hypotheses (recovered microbial DNA is from the presumptive source and recovered microbial DNA is from a source different from the presumptive one). In two case scenarios, Lindgren and colleagues [45] utilised genomic signatures from available sequence data in the calculation of evidence values. In both cases, the likelihood ratio approach was found to be very practical in evidence interpretation.

## Health system resilience and the potential contributions of national microbial forensic systems

The natural spread of infectious microbes during outbreaks and the intentional release and propagation of artificially engineered microbial agents are major global concerns, especially in resource-limited settings. Health system resiliency is weakened by outbreaks of infectious diseases and natural hazards. In these events, response strategies must be people-centred to prevent mistrust and undermining of the authority of government agencies serving as focal health players [17, 46]. The principles of leadership and command structure, surge capacity, and health workforce are critical during public health emergencies [47]. Leadership and command capacity refers to the existence of a clear and flexible command structure before the spread of infectious disease. Surge capacity refers to the extent to which a health system can call on human and capital resources to address increases in needed medical care. Lastly, health workforce capacity refers to the available trained and willing workforce to serve as medical and allied healthcare frontliners. A national microbial forensic system strongly contributes to the improvement of these key capacities. To the best of our knowledge, no country has legitimised a national microbial forensic system that caters to biological attacks and natural hazards. However, the US NBFAC and the Australian Federal Police (AFP) may be considered early adopters.

The NBFAC, under the US Department of Homeland Security, is the lead federal facility for forensic analysis and evidence derivation following a biological attack. It provides dedicated staff, laboratories, equipment, and procedures to conduct forensic analysis to carry out attribution of biocrimes or bioterrorism events. The NBFAC first opened in May 2004 and was equipped with BSL 2 and BSL 3 laboratories. After 6 years, the NBFAC laboratories were permanently transferred to Maryland and were housed on the National Interagency Biodefense Campus [48]. The AFP, on the other hand, is a model combining integrated intelligence and forensics in approaching threats posed by bioterrorism. Recognising that the law enforcement community had virtually no experience and expertise in microbiology, the AFP established a programme to build in-house capacity on basic microbiology. It also promoted partnerships with diagnostic and specialty laboratories for investigations of a more intensive nature. The AFP is capable of screening and identifying potentially infectious biological agents. It is also capable of applying standard forensic procedures from the crime scene up to the laboratory. The AFP works in close collaboration with the Australian Chemical, Biological, Radiological, and Nuclear Data Centres as well as with international partners in the UK, Canada, and the USA [49].

**Figure 1.**
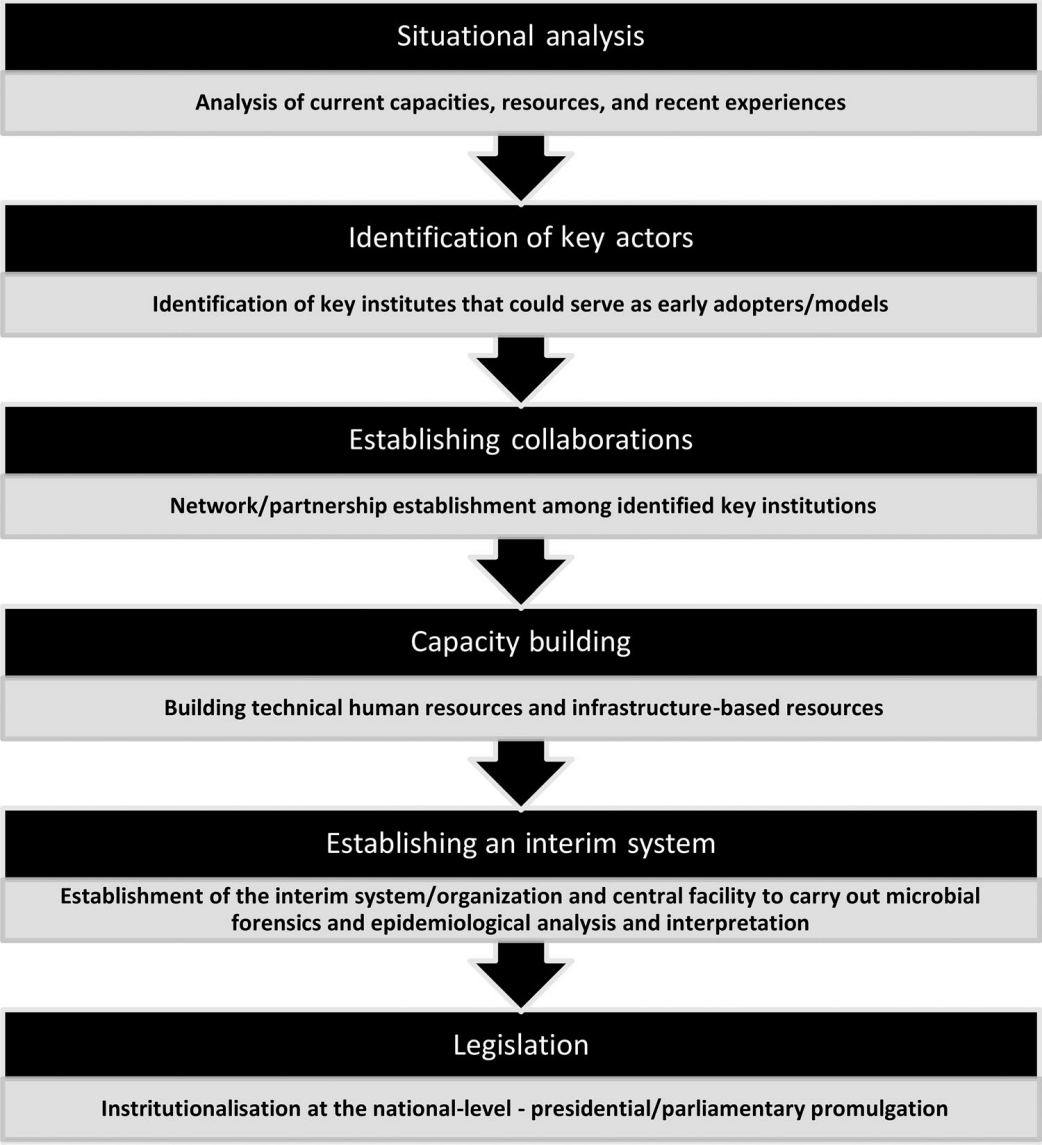
Key activities in establishing a national microbial forensic system.

Lifting from the shared domains of microbial forensics and molecular epidemiology as well as from the NBFAC and AFP experiences, a basic roadmap in establishing national microbial forensic systems is presented below (Figure 1). Any country that intends to establish a central system catering to microbial forensic and epidemiological investigations must initially analyse the current situation and identify the existing institutes that could serve as key players in the initiative. A situational analysis provides baseline information on the current capacities of a country as well as limitations due to available resources (e.g. national budget, human capital, and existing organizational structures). It will be costly to establish national microbial forensic systems for countries with limited to no resources and without pre-systems or early adopters already doing microbial forensic work. An initial assessment will also contribute to the identification of gaps pertinent to surge capacity and health workforce capacity (i.e. low numbers of trained scientists and absence of microbial forensic laboratories) (Table 4).

**Table 4.** Challenges in establishing national microbial forensic systems.

Limitations	Potential problems/challenges
**Financial resources**	Expensive for countries with no resources and no pre-systems/early adopters for forensic analysis
**Natural ubiquity, high genetic diversity, and rapid evolutionary adaptability of microbes**	Sufficient biobanking facility to maintain and preserve all microbial strains encountered during investigations Continuous evolution and adaptation of microbes resulting in emerging and re-emerging pathogens
**Methodological and analytical requirements**	Absence of robust molecular typing systems Choice of informative analytical tools
**Infrastructure and human resource requirements**	Low number of trained scientists and technicians Absence of microbial forensics-ready laboratories

Ultimately, strategies will hinge on the detailed context provided by baseline situational analyses. From initial situationers, key institutes that could adopt a microbial forensics framework should be identified and supported with additional funding and infrastructure. Focal scientists should be invited to serve as primary consultants in establishing a state-specific national microbial forensic system. Forging partnerships in realising capacities and systems for microbial forensic work is exemplified by the AFP experience, which paved the way for the development and sustenance of its capacities [49]. Eventually, the need for both an interim organisation and a centralised processing facility will improve in time, following the increased frequencies of cases of disease outbreaks, biocrimes, or bioterrorism. Through legislation, this interim organisation will serve as a precursor to a more independent unit that would be recognised at the national level as the leader for microbial forensics and molecular epidemiology work. With the increasing threats of biological attacks and disease outbreaks, the NBFAC experience is a testament to the successful establishment of microbial forensic systems over time [48]. The steps of the roadmap (Figure 1) corresponds to the thematic grounding of a conceptualised health system resilience framework by Haldane *et al.* [50]. The criticality of multi-sectoral collaboration and the harnessing and sustenance of current resources set things in motion towards establishing microbial forensic systems that consider local conditions and resources.

## Challenges in establishing microbial forensic systems at a national level

Budowle *et al.* [19] discussed two challenges that must be addressed to ensure the functionality of any national microbial forensic system. First is the need for robust and rational typing systems (Table 4). For the longest time, molecular typing has been used in epidemiological studies. However, its discriminatory power is low. With the genomic plasticity of infectious microbes, selecting the most informative analytical approach is critical. Another gap that needs to be addressed is choosing the most informative analytical approach and tool to be employed. Infectious microbes can be typed and further studied using multiple data sets (e.g. WGS, gene expression profiles, and peptide mass fingerprints). Hence, it is important to identify which approach and data should be used. Considering a hierarchy in terms of utilising these data as evidence is also needed. Analyses of omics data will require validated algorithms that must consider population-level factors such as clonal inheritance, inheritance through sexual reproduction, gene conversion, recombination, and horizontal gene transfer [18].

Another major challenge in developing a national microbial forensic system is microbial species diversity (Table 4). Microbial forensic analyses are often laborious and information-heavy due to the high diversity of microbes [11]. Numerous microbes can be considered as candidate agents in microbial forensic cases. This inherent problem aggravates the challenges of species typing and data analysis. The diversity of infectious microbes and the fact that most of them are not yet adequately identified are critical challenges that a national microbial forensic system will eventually have to address. Sufficient biobanking facilities and robust typing systems are needed. In terms of operation, another limitation is the cost of technical expertise and material needs in the wet laboratory (Table 4). Even though omics technologies are becoming cheaper, there would still be instances that require capture and comparisons using big data sets (i.e. WGS and metagenomic data). Cases that require big data involve microbial identities that demonstrate high degrees of phylogenetic relatedness, where differentiation can only be achieved through genome-level comparisons. Data capture does not only require technical expertise but also well-equipped laboratories that produce genomic, transcriptomic, or proteomic data. Trained scientists and technicians make use of expensive equipment and technologies [e.g. nucleic acid sequencing, Liquid Chromatography-Mass Spectrometry (LC-MS)], to produce data needed to understand microbial behaviour in biological attacks and disease outbreaks.

In addition to these technical challenges, country-specific health policies must be based on three major goals: (1) implementation of a standard guide for outbreak/disease management that focuses on pathogen identification, prevention, treatment, and control of transmission across communities; (2) effective communication and reporting channels from field units up to the national government agency in charge of public health; and (3) effective and timely response system to ensure functional delivery of healthcare services to the affected population. Notably, LMICs are prone to infectious diseases due to weak diagnostic capacities and rampant use of counterfeit drugs [51, 52]. The prevention of diseases through natural causes or artificial means such as biocrimes or bioterrorist attacks is part of public health. Hence public infrastructure must be made available to address such health emergencies [53].

Initiating a national microbial forensic system will require preliminary recommendations: (1) establishing partnerships among the law enforcement/police, public health, disaster resilience, and agriculture sectors; (2) creating a national computerised network that will track outbreaks and episodic cases; (3) capacitating field epidemiologists, veterinary and medical microbiologists, bioinformaticians, and other allied health and medical professionals in the technical elements of microbial forensics as it parallels regular epidemiologic procedures; and (4) institutionalising a national policy detailing the standard conduct of identification and characterisation of biological agents responsible for infectious diseases. Justifying the need to realise these initial recommendations is a daunting task as the expected outputs and outcomes require substantial funding. In most cases, a government’s health budget is pooled singly to finance investments for health services that are either preventive at the population health level or curative at the individual health level. The allocation of a government’s health budget is influenced by several factors such as existing policy commitments and national health objectives (e.g. efficiency and equity) [54]. In both HICs and LMICs, it has been observed that the total health expenditures for curative services tend to surpass those intended for prevention [55, 56] and this may result in various consequences, ultimately different health outcomes. Unfortunately, the strategy of focusing on individual diseases/conditions has also been observed to produce inconsistent results in terms of health outcomes and quality of life. As a disease-preventing investment at the population health level, a unified system catering to microbial forensics and molecular epidemiology is a timely cause to be prioritised.

With its expected high-level impacts on health and non-health dimensions of human life, a unified microbial forensic/epidemiological system is a viable option that can be started with small steps. Lobbying for the creation of a national microbial forensic system must consider prevailing social and political priorities and amid re-emerging and emerging disease agents, now is the best time for scientists and consultants to make the case for it. Given the ongoing global SARS-CoV-2 pandemic, health leaders and policymakers must seriously consider the urgency of adopting a national strategy that uses microbial forensic and epidemiological principles to strengthen the health system.

## Conclusions

Gaps in understanding infectious diseases should prompt health systems all over the world to prioritise the establishment of national microbial forensic systems. Conceptual and methodological similarities of microbial forensics and molecular epidemiology can contribute to strengthening a health system’s resiliency and capacity to address public health emergencies. Advancing microbial forensics through a systemic approach will entail the recognition and resolution of challenges related to financing, microbial diversity and adaptability, analytical tools, infrastructure, and human requirements. With the current state of global health, operationalising a national microbial forensic system is a priority. National microbial forensic systems are envisioned to improve surveillance and containment of re-emerging and emerging infectious diseases.
